# A dual tag system for facilitated detection of surface expressed proteins in *Escherichia coli*

**DOI:** 10.1186/1475-2859-11-118

**Published:** 2012-09-03

**Authors:** Johan Jarmander, Martin Gustavsson, Thi-Huyen Do, Patrik Samuelson, Gen Larsson

**Affiliations:** 1 School of Biotechnology, Division of Bioprocess Technology, Royal Institute of Technology, KTH, Stockholm, SE, 106 91, Sweden; 2 Institute of Biotechnology, Vietnamese Academy of Science and Technology, VAST, 18-Hoang Quoc Viet, Hanoi, Cau Giay, Vietnam

**Keywords:** AIDA, Surface expression, Autotransport, *Escherichia coli*, Proteolysis, Detection tag

## Abstract

**Background:**

The discovery of the autotransporter family has provided a mechanism for surface expression of proteins in laboratory strains of *Escherichia coli*. We have previously reported the use of the AIDA-I autotransport system to express the *Salmonella enterica* serovar Enteritidis proteins SefA and H:gm. The SefA protein was successfully exposed to the medium, but the orientation of H:gm in the outer membrane could not be determined due to proteolytic cleavage of the N-terminal detection-tag. The goal of the present work was therefore to construct a vector containing elements that facilitates analysis of surface expression, especially for proteins that are sensitive to proteolysis or otherwise difficult to express.

**Results:**

The surface expression system pAIDA1 was created with two detection tags flanking the passenger protein. Successful expression of SefA and H:gm on the surface of *E. coli* was confirmed with fluorescently labeled antibodies specific for the N-terminal His_6_-tag and the C-terminal Myc-tag. While both tags were detected during SefA expression, only the Myc-tag could be detected for H:gm. The negative signal indicates a proteolytic cleavage of this protein that removes the His_6_-tag facing the medium.

**Conclusions:**

Expression levels from pAIDA1 were comparable to or higher than those achieved with the formerly used vector. The presence of the Myc- but not of the His_6_-tag on the cell surface during H:gm expression allowed us to confirm the hypothesis that this fusion protein was present on the surface and oriented towards the cell exterior. Western blot analysis revealed degradation products of the same molecular weight for SefA and H:gm. The size of these fragments suggests that both fusion proteins have been cleaved at a specific site close to the C-terminal end of the passenger. This proteolysis was concluded to take place either in the outer membrane or in the periplasm. Since H:gm was cleaved to a much greater extent then the three times smaller SefA, it is proposed that the longer translocation time for the larger H:gm makes it more susceptible to proteolysis.

## Background

Surface expression of recombinant proteins was first described more than 25 years ago [1,2]. Systems for surface expression in both Gram-negative and Gram-positive bacteria have been reported with a broad range of applications in molecular biology, biochemistry, biotechnology, microbiology and vaccinology [3-7]. The Gram-negative bacterium *Escherichia coli* has historically been one of the most extensively used hosts for recombinant protein production [8]. For surface expression of heterologous proteins, Gram-positive bacteria should theoretically be simpler to use than Gram-negative, as the expressed protein needs to be translocated over only one cell membrane instead of the two required with Gram-negative bacteria. However, since there is extensive documented knowledge regarding the genetics, growth and protein production of *E. coli*, it is an attractive platform also for surface expression applications. Laboratory strains of *E. coli* have the additional advantage that they lack inherent surface protein transporters, so there is less background of natural proteins on the cell surface and in the medium.

The discovery of the type V protein secretion pathway and of the autotransporter family [9] has provided great opportunities for surface expression of proteins in *E. coli*. The surface translocation system of AIDA-I, the adhesin involved in diffuse adherence of enteropathogenic *E. coli*, has for example been successfully transferred to laboratory strains of *E. coli*[10] and used for expression of recombinant proteins [11], enzymes [12] and enzyme inhibitors [13] on the cell exterior. Autotransporters have a conserved sequence organization consisting of an N-terminal signal peptide followed by the passenger protein, a linker region and a C-terminal translocation unit [14]. In the translocation process, the signal peptide is cleaved off after a Sec-mediated passage over the inner membrane. The widely accepted hypothesis is that the C-terminal translocation unit then inserts itself into the outer membrane and forms a β-barrel pore through which the linker and passenger protein pass and become surface exposed.

We recently reported the display of the *Salmonella enterica* serovar Enteritidis (*S*. Enteritidis) flagellar protein H:gm and fimbrial protein SefA on the surface of *E. coli*, using the AIDA-I autotransport system [15]. The proteins were expressed as fusions with an N-terminal His_6_-tag to remove the need for protein specific antibodies for detection of the passenger. In the vector that was used, the cleavage site for releasing the natural passenger from the cell surface had been removed, and the fate of the expressed proteins were to stay covalently bound to the translocation unit. The aim of that study was to explore the possibility of using *E. coli* as a platform for presenting surface-exposed antigens. SefA was successfully displayed on the cell surface, but the orientation of H:gm in the outer cell membrane could not be resolved due to cleavage and loss of the His_6_-tag. The hypothesis that both fusion proteins were facing the cell exterior could not therefore be experimentally confirmed.

The present work aimed to create a vector with a dual tag surface expression system, where two tags flank the passenger, in order to increase the possibility of detecting proteins that are sensitive to proteolysis or difficult to translocate. We wished to use this vector to confirm the previous hypothesis that the H:gm fusion protein did indeed have the correct orientation in the outer membrane, establishing the vector as an improved surface expression analysis tool. As the new vector leads in principle to the expression of a new fusion protein, we also wished to compare the relative expression levels with those obtained with the previously used surface expression system. SefA was chosen as the model protein for this comparison as it had earlier been successfully expressed.

## Results

### Construction of the surface expression system

**Figure 1:**
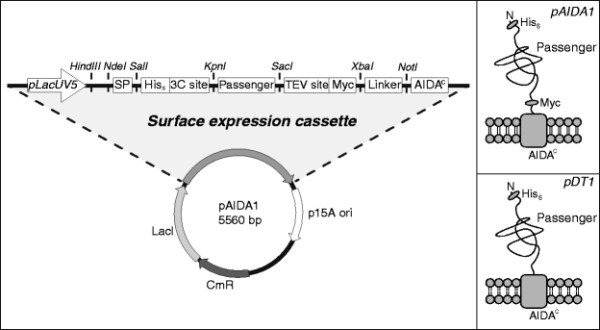
**Schematic representations of pAIDA1 and the surface expressed fusion proteins of pAIDA1 and pDT1.** Left: The surface expression vector pAIDA1 based on the AIDA-I autotransporter. Expression is under the control of the lacUV5 promoter, and two detection tags (His_6_ and Myc) with adjacent protease cleavage recognition sites (for 3C and TEV) flank the passenger protein. Top right: Matured fusion protein at the cell surface from vector pAIDA1. Bottom right: Matured fusion protein at the cell surface from vector pDT1.

The vector constructed in this work was named pAIDA1 and led to the expression of a fusion protein that holds a 5 kDa signal peptide, a 5 kDa linker region and a 47 kDa AIDA^C^ translocation unit, for a total size of 63 kDa not including the passenger (Figure 1 left, DNA sequence in Additional file 1). After the signal peptide had been cleaved off, the fusion protein had a mature size of 58 kDa in the outer membrane. The previously used surface expression vector pDT1 [15] had functional units of the same size and translated a protein of 62 kDa with a mature size of 57 kDa in the membrane. The main feature of pAIDA1 is that the translated fusion protein contains two detection tags flanking the passenger on each side, compared to the protein product of pDT1, which has only one tag on the passenger’s N-terminal side (Figure 1 right).

In pAIDA1, a His_6_-tag was placed on the N-terminal side of the passenger, as was previously done in pDT1. The redundant nucleotides found in the gap between the tag and the passenger were however removed, and a cleavage site for the recombinant type 14 3C protease from human rhinovirus (HRV 3C, aa seq: LEALFQGP) was instead introduced in this space. On the C-terminal side of the passenger, a Myc-tag [16] was introduced. In this case, a protease cleavage was also placed between the tag and the passenger, for the Tobacco etch virus protease (TEV, aa seq: ENLYFQG). Since only relative surface expression levels can be measured by the detection of tags, the introduction of protease cleavage sites makes it possible to develop a method for quantifying surface expression through conventional protein analysis.

The natural promoter of AIDA-I, *aidA*, that was used in pDT1, transcribes mRNA constitutively and is regulated by a mechanism that is not well understood [17]. In pAIDA1, it was therefore replaced by the lacUV5 promoter, which is a well-studied, inducible promoter that can be successfully titrated in *lacY* negative *E. coli* strains to desirable expression levels [18]. In order to facilitate the modification of the novel vector, unique restriction sites were placed between each component in the expression cassette so that each unit could be manipulated independently.

### Surface expression of proteins

*E. coli* cells harboring pAIDA1 and pDT1 expressing the fusion proteins of SefA and H:gm were cultivated in batch, and cell samples from the logarithmic growth phase were removed for analysis. The mature fusion proteins had the following theoretical sizes in the outer membrane; pDT1-SefA: 71 kDa, pDT1-H:gm: 110 kDa, pAIDA1-SefA: 72 kDa and pAIDA1-H:gm: 111 kDa. By using His_6_- and Myc-tag-specific conjugated antibodies, the tags could be detected on the cell surface and the expression analyzed with flow cytometry. The fluorescence of 10 000 events (cells) was recorded, giving a normal distribution of the fluorescence signal. The mean fluorescence thus corresponds to the average amount of expressed protein per cell analyzed.

**Figure 2:**
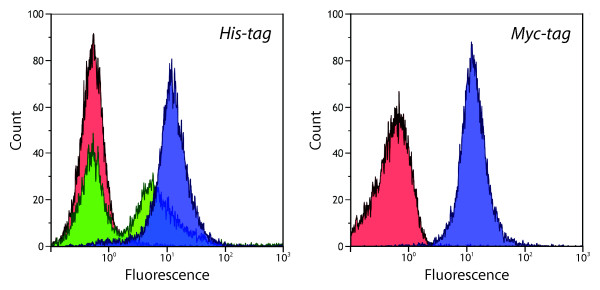
**Flow cytometry histograms of expressed SefA on the surface of *E. coli*.** Left: Detection of His_6_ tag by the THE^TM^ His Tag [FITC] antibody. Negative control (red) consisting of cells lacking vector showed a mean fluorescence of 0.6. Cells harboring pDT1-SefA (green) showed a mean fluorescence of 10.5 in the positive population. Cells harboring pAIDA1-SefA (blue) show a mean fluorescence of 15.3. Right: Detection of Myc tag by Anti-c-Myc [SureLight® Allophycocyanin] antibody. Negative control (red) showed a mean fluorescence of 0.6 and cells harboring pAIDA1-SefA (blue) show a mean fluorescence of 15.4.

The surface expression of SefA from the two different vectors was first analyzed in order to determine the relative expression level of pAIDA1 in relation to that of the previously used system. Cells expressing SefA from pDT1 showed a greater fluorescence then a negative control (*E. coli* cells lacking vector), when detected with an anti-His_6_ antibody (Figure 2). The fluorescence was divided into two distinct peaks with narrow distributions, each holding approximately 50% of the total amount of cells. One of the peaks had the same mean fluorescence as the negative control of 0.6, and the other had a 17 times higher mean fluorescence of 10.5, showing that the tag was detected in only half the cell population. When pAIDA1 was used as the expression vector for SefA, a single narrow peak was seen with a mean fluorescence of 15.3, approximately 25 times higher than that of the negative control and 1.5 times higher than that of pDT1-SefA. The His_6_-tag was thus detected on all cells harboring pAIDA1-SefA.

The Myc-tag detection was thereafter tested for the fusion protein of pAIDA1-SefA, using an anti-Myc antibody. The resulting fluorescent response was similar to that obtained for the His_6_-tag (Figure 2), with a narrow distribution of the fluorescence with a mean value of 15.4. This is also about 25 times greater than the value for the negative control at 0.6, confirming the successful binding of the antibody to the Myc-tag.

**Figure 3:**
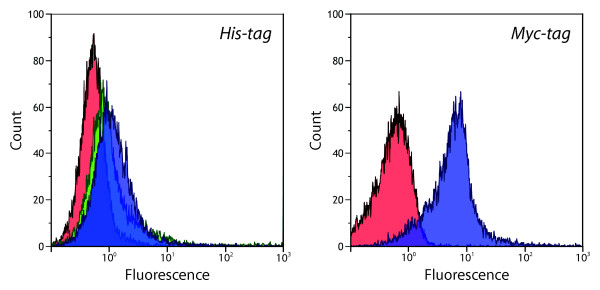
**Flow cytometry histograms of expressed H:gm on the surface of *E. coli*.** Left: Detection of His_6_-tag by the THE^TM^ His Tag [FITC] antibody. Negative control (red) consisting of cells lacking vector showed a mean fluorescence of 0.6. Cells harboring pDT1-H:gm (green) showed a mean fluorescence of 1.4. Cells harboring pAIDA1-H:gm (blue) show a mean fluorescence of 1.5. Right: Detection of Myc-tag by Anti-c-Myc [SureLight® Allophycocyanin] antibody. Negative control (red) showed a mean fluorescence of 0.6 and cells harboring pAIDA1-H:gm (blue) show a mean fluorescence of 7.6.

Surface expression of H:gm was investigated after it had been confirmed that the tags in the new fusion protein could be detected. When this fusion protein was expressed from pTD1, detection with the anti-His_6_ antibody gave rise to only a small increase in the fluorescence, 1.4 compared to 0.6 for the negative control (Figure 3). Expression of the H:gm fusion protein from pAIDA1 gave a comparable mean fluorescence of 1.5. The fluorescence of H:gm was thus 10 times lower than that of SefA for both systems, showing a lower presence of His_6_-tags on the cell surface. The anti-Myc antibody was then used to detect the Myc-tag of the pAIDA1-H:gm fusion protein. The resulting fluorescence showed a single, narrow distribution with a mean value of 7.6, about 12 times higher than that of the negative control (Figure 3). This demonstrates that the Myc-tag also was present on the surface of cells expressing pAIDA1-H:gm.

### Cellular distribution and protein size

**Figure 4:**
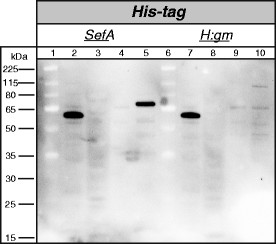
**Western blot showing distribution of SefA and H:gm from pDT1 in cellular fractions.** Detection through an anti-His_6_ antibody conjugated with horseradish peroxidase. Lanes 1 and 6: Size marker. Lanes 2 and 7: Outer membrane fraction of pAIDA1 without passenger (positive control). Lane 3: pDT1-SefA soluble fraction. Lane 4: pDT1-SefA inner membrane fraction. Lane 5: pDT1-SefA outer membrane fraction. Lane 8: pDT1-H:gm soluble fraction. Lane 9: pDT1-H:gm inner membrane fraction. Lane 10: pDT1-H:gm outer membrane fraction.

To understand the fate and localization of the fusion proteins during translocation, the soluble inner membrane and outer membrane protein fractions were isolated and analyzed by Western blot. The membrane fractions of pDT1-SefA and pDT1-H:gm were blotted and detected with an anti-His_6_ antibody conjugated to horseradish peroxidase (HRP) (Figure 4). The fusion protein of pAIDA1 without a passenger was used as a positive control. In the case of pDT1-SefA, a strong band was visualized in the outer membrane fraction between the 65 and 80 kDa markers, in good agreement with the fusion protein’s mature size of 71 kDa. In the case of pDT1-H:gm, only a weak band could be seen in the outer membrane fraction slightly below the 115 kDa marker, probably the mature fusion protein which has a size of 110 kDa. The positive control pAIDA1 encodes a mature fusion protein of 58 kDa, which agrees well with the position of the strong band seen slightly below the 65 kDa marker. These results confirm those previously obtained for the expression of pDT1-SefA and pDT1-H:gm [15].

**Figure 5:**
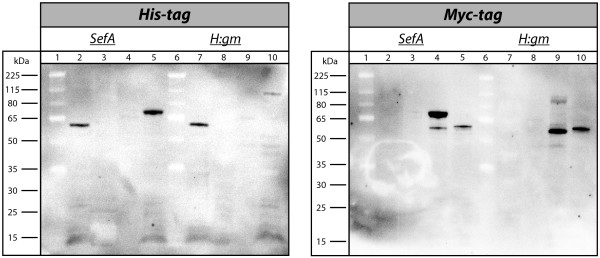
**Western blot showing distribution of SefA and H:gm from pAIDA1 in cellular fractions.** Left: Detection through an anti-His_6_ antibody conjugated with horseradish peroxidase. Lanes 1 and 6: Size marker. Lanes 2 and 7: Outer membrane fraction of pAIDA1 without passenger (positive control). Lane 3: pAIDA1-SefA soluble fraction. Lane 4: pAIDA1-SefA inner membrane fraction. Lane 5: pAIDA1-SefA outer membrane fraction. Lane 8: pAIDA1-H:gm soluble fraction. Lane 9: pAIDA1-H:gm inner membrane fraction. Lane 10: pAIDA1-H:gm outer membrane fraction. Right: Detection through an anti-Myc antibody conjugated with horseradish peroxidase. Lanes 1 and 6: Size marker. Lane 2: pAIDA1-SefA soluble fraction. Lane 3: pAIDA1-SefA inner membrane fraction. Lane 4: pAIDA1-SefA outer membrane fraction. Lane 5 and 10: Outer membrane fraction of pAIDA1 without passenger (positive control). Lane 7: pAIDA1-H:gm soluble fraction. Lane 8: pAIDA1-H:gm inner membrane fraction. Lane 9: pAIDA1-H:gm outer membrane fraction.

The fusion proteins of pAIDA1-SefA and pAIDA1-H:gm were thereafter blotted and detected with anti-His_6_ and anti-Myc antibodies, both conjugated to HRP (Figure 5). For the blot detected with the anti-His_6_ antibody, the profile was almost identical to that of pDT1. Both fusion proteins were found exclusively in the outer membrane. For SefA there was a strong band, while the corresponding band for H:gm was weak. Detection from the anti-Myc antibody also revealed bands in the outer membrane fractions. For SefA, two bands were seen in this fraction, a strong band at the mature size of 72 kDa, but also a weaker band slightly smaller than the positive control at 58 kDa. For H:gm, a weak band appeared at the anticipated size of 111 kDa, but a strong band was also visible at the same size as that of degraded SefA. This means that both fusion proteins had to some degree been cleaved on the N-terminal side of the Myc-tag, although the cleavage was more extensive for the fusion protein of H:gm.

## Discussion

With regard to the surface expression of the SefA fusion protein, it was demonstrated that the anti-His_6_ antibody gave a mean fluorescence that was 1.5 times higher for pAIDA1 than for pDT1. Thus not only was the new system successfully displaying this protein on the surface, it also indicated a 50% higher expression level than the previously used system. In addition, pDT1 displayed two subpopulations, where the His_6_-tag could be detected on the surface of approximately half the cells. The reason for this is presently unknown. In the case of pAIDA1, all the cells displayed the tag, making the total surface expression level greater for pAIDA1 than for pDT1.

The distance of the C-terminal Myc-tag from the cell surface and the passenger protein could be of great importance for the success of the interaction with the associated antibody. If it is too close to the passenger protein, the passenger may perhaps screen the tag and sterically obstruct the antibody from binding to its target. If the tag is too close to the cell surface, other surface proteins might block the antibody. Nevertheless, it is desirable not to have an excessively long fusion protein. We chose to place the Myc tag adjacent to the TEV protease cleavage site, giving it a distance to the passenger that was approximately the same as for the His_6_-tag in pDT1, a tag that had been successfully detected in the past. Since the Myc-tag of the SefA fusion protein was successfully detected, we concluded that the chosen location did not lead to any significant steric limitation.

Detection of the His_6_-tag within the H:gm fusion protein gave rise to only a small increase in the fluorescence, whether expressed from either pDT1 or pAIDA1. This indicates that the majority of the His_6_-tags are either being cleaved off or getting stuck during translocation. Since the Myc-tag of pAIDA1 could however be detected with a significant increase in the fluorescence, we conclude that the H:gm fusion protein was indeed expressed on the cell surface, confirming our previous hypothesis. We can also say that the protein has been subjected to major proteolytic cleavage on the N-terminal side of the Myc-tag.

Western blot analysis allowed us to determine the protein’s cleavage pattern. That a single degradation product, slightly smaller in size than the positive control of 58 kDa, could be found both for the fusion protein of SefA and for H:gm, suggests that they have been cleaved in the same position. The cleavage has probably taken place close to the C-terminal end of the passenger, which would generate a fragment with a size in the range of 54–57 kDa. This information allows us to better understand the cause of the proteolytic problem, which may be used to improve the performance.

Since we identified that the cleavage took place from the N-terminal side of the fusion proteins, while still being able to detect them in the outer membrane, we can exclude the cytoplasm as a possible compartment for the proteolysis. The N-terminal end of the translated fusion protein harbors the signal peptide, which is necessary for translocation over the inner membrane through the Sec system. It has been shown that autotransporters will not be directed to the Sec translocon if the signal peptide is missing [19], and no protein would be found in the outer membrane if it was cleaved off prior to translocation. The proteolysis must therefore take place either in the periplasm or at the cell surface.

If the cleavage is localized to the outer membrane, it is probably due to the action of an outer membrane protease. The gene encoding for the outer membrane protein T (OmpT) has been deleted in the *E. coli* strain used in this study. This protease mainly cleaves between dibasic amino acid residues and it has previously been shown to degrade some recombinant proteins [20] and surface expression has been improved in its absence [17]. With OmpT deleted, a candidate for outer membrane proteolysis is the outer membrane protein P (OmpP), a homologue to OmpT, that exhibits very similar substrate specificity [21]. There are however several reasons to believe that OmpP is not responsible for the proteolysis. OmpP is an F-episome-encoded protein [22] and the strain used in this study is F^-^ so that, unless the episome has integrated spontaneously into the chromosome in the past, its DNA should not be present. Further, we have not been able to amplify the OmpP gene from the strain (data not shown), and this is probably due to its absence. Lastly, there are no appropriate cleavage sites for this protease within the fusion protein that would generate a fragment of the length seen on the Western blots.

It is therefore more probable that the proteolysis takes place in the periplasmic area. It might be expected that a larger protein would remain unfolded in the periplasmic space for a longer period of time than a smaller counterpart, and that this would make it more vulnerable to periplasmic proteases. This could explain the extensive cleavage of H:gm, which is three times longer (507 aa to 165 aa) than the less affected SefA.

It could also be so that the proteins become partly folded in the periplasm, and that this inhibits translocation over the outer membrane. As the C-terminal β-barrel is first inserted into the outer membrane, followed by translocation of the linker and then the passenger through the pore, premature folding might make it sterically impossible for the protein to enter the pore. Although autotransporters have been shown to tolerate periplasmic folding of the passenger to some degree, the β-barrel provides only a very narrow channel for translocation [23]. The crystallized β-barrel structure formed from NalP, an autotransporter of *Neisseria meningitides*, is for example only wide enough to hold two polypeptide strands at a time.

**Figure 6:**
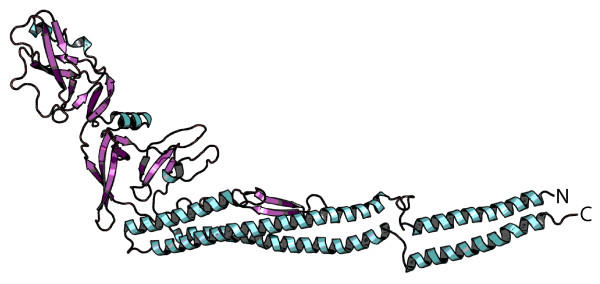
**Tertiary structure of H:gm.** Atomic model of the *Salmonella enterica* flagellar protein H:gm by electron cryomicroscopy [PDB: 1UCU] drawn in PyMOL. The protein consists of 494 aa and forms a tubular tertiary structure with the N- and C-terminals positioned close to each other.

From the tertiary structure of H:gm (Figure 6), it is evident that the N- and C-terminals of the protein are close to each other. Premature folding of this protein could therefore potentially generate a bulky structure incapable of translocation. It is intuitive to believe that the translocation of small proteins such as SefA will be less affected by periplasmic folding, as their partly folded forms are probably still small enough to fit inside the β-barrel. If a passenger protein does become trapped inside the periplasm, it will inevitably be cleaved by one of the proteases residing there, such as Mi, Tsp, Pitrilysin or DegP, allowing the Myc-tag to pass though the pore and become exposed to the cell exterior.

## Conclusions

The novel dual tag surface expression vector pAIDA1 was successfully constructed and used to express the proteins SefA and H:gm on the surface of *E. coli*. The expression levels were comparable to or higher than those achieved with the previously used vector. The extended detection possibilities offered by the new vector allowed us to verify the hypothesis that proteolysed H:gm in the outer membrane was expressed on the cell surface. The vector also made a detailed analysis of cleaved proteins possible, and single degradation products were found for both SefA and H:gm. These appeared to be of the same size, indicating a specific cleavage in the fusion protein close to the C-terminal end of the passenger protein.

Since the proteins had been cleaved from the N-terminal but were nonetheless present in the outer membrane, it was concluded that the compartment of proteolysis was either the outer membrane or the periplasmic area. Since an OmpT negative production strain that probably also lacks OmpP was used, the periplasm was considered to be the more probable of the two. There may be a correlation between protein length and proteolysis, making longer proteins more susceptible to periplasmic proteases. This could be due to a longer unfolded exposure in the periplasm or to more extensive problems with premature folding.

In summary, the dual tag system presented in this work allowed us to study surface expression from the AIDA-I autotransporter in greater detail than was previously possible. It contributed to an increased understanding of the proteolytic problem during protein translocation over the *E. coli* double membrane structure, and is thus a more comprehensive research tool. A natural continuation of this study is to isolate the source of the proteolysis, and the results obtained here will greatly help in doing so.

## Methods

### Vectors

The vectors pDT1-SefA and pDT1-H:gm are previously constructed derivatives of pBR322 that holds the natural AIDA-I promoter and the AIDA-I specific signal peptide [15]. The vectors express a fusion protein consisting of the passenger (SefA or H:gm) with an N-terminal His_6_-tag, a linker region made up of the 54 C-terminal aa of the natural AIDA-I passenger, and lastly the AIDA-I translocation unit (AIDA^c^). The vectors also contain an ampicillin resistance gene. The sizes of the vectors were 5977 bp for pDT1-SefA and 7066 bp for pDT1-Hgm.

For the construction of pAIDA1-SefA and pAIDA1-H:gm, standard recombinant DNA techniques were used [24]. Fermentas supplied restriction enzymes, ligase and the plasmid miniprep kit, whilst GE Healthcare supplied the PCR DNA and gel band purification kit. Primers and sequencing services were ordered from Eurofins MWG Operon. Primers used are listed in Table 1.

**Table 1:** Primers used for amplification of the components in pAIDA1

**Oligo name**	**Sequence**
AIDA_forw_HindII	GAT CCC GAC ACA GGA AAG C
AIDA_rev_BclI	CTC TGA TCA TTA TCA GAA GCT GTA TTT TAT CC
SefA_forw_KpnI	ACT GGT ACC GTG ATC ATA GCT GGC TTT GTT GGT AAC AAA GC
SefA_rev_SacI	ACT GAG CTC GTT TTG ATA CTG CTG AAC GTA G
H:gm_forw_delKpnI	AAT CTA CTG CTG GCA CCG CTG AAG
H:gm_rev_delKpnI	CGG TTG ACC TCT TTA AGA CCA CTG
H:gm_forw_KpnI	AAA AGG TAC CGC ACA AGT CAT TAA TAC AAT CAG CCT G
H:gm_rev_SacI	AAA AGA GCT CAC GCA GTA AAG AGA GGA CGT TTT

The vector pAIDA1 was constructed from pKM1D; a pACYC184 derivative that holds a p15A ori, a multiple cloning site under the control of the lacUV5 promoter, the lacI repressor gene and a chloramphenicol resistance gene. A gene fragment containing the AIDA-I signal peptide, a redesigned multiple cloning site for passenger insertion, the linker region and the AIDA-I translocation unit was synthesized by Eurofins MWG Operon, and then cloned into pUC57. The signal peptide, the linker and the translocation unit were identical to those in pDT1. The gene fragment was amplified with DreamTaq DNA polymerase (Fermentas) using the primers AIDA_forw_HindIII and AIDA_rev_BclI, and subsequently digested with the restriction enzymes HindIII and BclI, while pKM1D was cleaved with HindIII and BamHI. The digested vector and gene fragment were after gel purification ligated, yielding the new vector pAIDA1. The insertion was verified by DNA sequencing. The size of the final vector was 5560 bp.

Plasmid pAIDA1-SefA was constructed by amplification of SefA from a pET32 plasmid by DreamTaq DNA polymerase using the primers SefA_forw_KpnI and SefA_rev_SacI. After digestion with the restriction enzymes KpnI and SacI of SefA and pAIDA1, and subsequent gel purification and ligation of the gene fragments, pAIDA1-SefA was created. This vector had a size of 5986 bp.

By amplification of the entire plasmid pDT1-H:gm with Phusion polymerase (Finnzymes) using the primers H:gm_forw_delKpnI and H:gm_rev_delKpnI, a silent point mutation (774 T > C) was introduced into H:gm to remove a recognition site for the restriction enzyme KpnI (GGTACC). The resulting amplicon was ligated and served as a template for the amplification of H:gm with Phusion polymerase from the primers H:gm_forw_KpnI and H:gm_rev_SacI. Plasmid pAIDA1 and the amplified gene was digested with the restriction enzymes KpnI and SacI and, following gel purification, ligated into pAIDA1-H:gm. The size of the vector was 7066 bp.

### Bacterial strain and cultivation medium

The *E. coli* K12 strain 0:17 (sup^+^, F^-^) [25] with the OmpT gene deleted (0:17ΔOmpT) was the host used for comparing the surface expression of SefA and H:gm encoded from pDT1 and pAIDA1. The OmpT negative strain was chosen in order to reduce cleavage of surface expressed proteins [17]. The cultivation medium used was a minimal salt medium consisting of (per liter): (NH_4_)_2_SO_4_, 5 g; KH_2_PO_4_, 1.6 g; Na_2_HPO_4_·2H_2_O, 6.6 g; (NH_4_)_2_-H-Citrate, 0.5 g. Sterile glucose solution with a concentration of 500 g l^-1^ was added to the medium after sterilization to a final concentration of 10 g l^-1^. The medium was also supplemented with 1 ml l^-1^ trace element solution and 1 ml l^-1^ sterile 1 M MgSO_4_, both sterile filtered (0.2 μm, VWR collection). The trace element solution contained the following components (per liter): CaCl_2_·2H_2_O, 0.5 g; FeCl3·6H_2_O, 16.7 g; ZnSO_4_·7H_2_O, 0.18 g; CuSO_4_·5H_2_O, 0.16 g; MnSO_4_·4H_2_O, 0.15 g; CoCl_2_·6H_2_O, 0.18 g; Na-EDTA, 20.1 g. Antibiotics in the form of chloramphenicol for pAIDA1 and ampicillin for pDT1, were added to ensure plasmid stability in concentrations of 10 μg ml^-1^ and 100 μg ml^-1^, respectively.

### Cultivation procedure

*E. coli* cells harboring the plasmids pDT1-SefA, pDT1-H:gm, pAIDA1-SefA and pAIDA1-H:gm were taken from glycerol stocks stored at −80°C and used to inoculate sterile baffled shake flasks with a volume of 1 liter containing 100 ml of cultivation medium. The flasks were incubated overnight at 37°C with 180 rpm shaking. The following day, exponentially growing cells were transferred to sterile 5 l shake flasks containing 500 ml medium, giving an initial OD_600_ of approximately 0.1. The cells harboring plasmids pAIDA1-SefA or pAIDA1-H:gm were grown to an OD_600_ of 0.2, at which point they were induced with IPTG (Apollo scientific) at a concentration of 200 μM to start the synthesis of surface expressed proteins. The induced cells were incubated for another 4 h before harvesting and analysis of surface expression. At harvest, the OD_600_ was 4.65 for cells holding pAIDA1-SefA and 4.30 for cells holding pAIDA1-H:gm. Cells harboring the plasmids pDT1-SefA and pDT1-H:gm were harvested at an OD_600_ of 3. Samples taken for analysis with flow cytometry were diluted in saline solution (0.9% NaCl w/v) to an OD of 2 and thereafter mixed 1:1 with 50% w/w glycerol before being frozen at −80°C. The stability of the preparation has been tested in previous experiments [17].

### Cell growth

The optical density at 600 nm was measured to monitor cell growth. Samples were aseptically withdrawn from cultures, diluted in saline solution to an OD_600_ of approximately 0.1 and measured in a spectrophotometer (Genesys 20, Thermo scientific). Actual OD_600_ values were obtained by multiplication of the measured values with the dilution factor.

### Isolation of outer membrane proteins

Outer membrane proteins were isolated as previously described [26]. 500 ml cultures were harvested by centrifugation at 2500 rpm for 15 min at 4°C (J-6B Centrifuge, Beckman). The resulting cell pellets were washed using 140 ml of membrane isolation buffer (50 mM Tris–HCl, pH 7.5), followed by a second centrifugation (2500 rpm, 15 min, 4°C). Cell pellets obtained after washing were stored at 4°C overnight. The following day, the pellets were dissolved in 11 ml membrane isolation buffer and the cells were disintegrated in a French press high-pressure homogenizer (SLM Aminco). Non-disintegrated cells were removed by centrifugation (2500 rpm, 15 min, 4°C). The resulting supernatants were centrifuged (36000 g, 40 min, 4°C) in order to collect membrane-bound proteins as pellets. The supernatants containing soluble proteins were aliquoted into microcentrifuge tubes and frozen at −20°C until analysis.

1.9 ml membrane isolation buffer was used to wash the membrane pellet, followed by a second centrifugation (36000 g, 40 min, 4°C). The membrane pellet was then dissolved in 12.5 ml membrane isolation buffer containing 0.1% (v/v) sarcosyl (*N*-laurylsarcosine sodium salt solution, Fluka) and incubated for 60 min on a shaker table at 4°C in order to separate the inner and outer membrane proteins. After incubation, the suspension was centrifuged (36000 g, 40 min, 4°C) and the inner membrane proteins were obtained in the supernatant while the outer membrane proteins remained in the pellet fraction. The membrane pellet was dissolved using 14.5 ml isolation buffer containing 1% (v/v) Triton-X-100 (SigmaUltra, Sigma-Aldrich) and 5 mM EDTA. Finally, both the inner and outer membrane protein fractions were aliquoted and frozen at −20°C until analysis.

### SDS-PAGE and Western blot

Three parts of protein sample were mixed with one part of 4x SDS loading buffer (Loading buffer pack, Fermentas) and incubated at 105 °C for 15 minutes. 10 μl sample and 7.5 μl protein ladder (Spectra^TM^ multicolor broad range protein ladder, Fermentas) were loaded onto 10% NuPage Bis-Tris gels (Invitrogen) and electrophoresis was run for 75 min in MOPS running buffer (10.46 g l^-1^ MOPS, 6.06 g l^-1^ Tris, 1 g l^-1^ SDS, 0.3 g l^-1^ EDTA) at room temperature. Gels were then equilibrated with transblot buffer (2.93 g l^-1^ glycine, 5.81 g l^-1^ Tris–HCl, 0.37 g l^-1^ SDS, 20% v/w methanol) for 30 min followed by blotting to equilibrated PVDF membranes (Westran® S, Whatman) for 75 min at 25 V (Trans-blot® SD Semidry transfer cell machine, Bio-Rad). The membranes were blocked for more then 1 h in phosphate buffered saline (PBS, 9 g l^-1^ NaCl, 0.21 g l^-1^ KH_2_PO_4_, 0.726 g l^-1^ Na_2_HPO_4_, pH 7.4) containing 5% w/v non-fat milk powder (Semper) and then washed for 5 min in PBS containing 0.5 ml l^-1^ Tween 20 (PBST). The washed membranes were incubated for 1 h in PBST containing 1 g l^-1^ Bovine serum albumin (BSA) and 0.5 μg ml^-1^ antibody (THE^TM^ His Tag [HRP] or THE^TM^ cMyc Tag [HRP], GenScript). The membranes were then washed four times for 5–10 min in PBST. Detection was achieved by addition of Amersham^TM^ ECL^TM^ Prime Western blotting reagent (GE Healthcare) to the membranes, and after 5 min of incubation the chemiluminescence was recorded on a Molecular Imager® ChemiDoc^TM^ XRS + (Bio-Rad).

### Labeling for flow cytometer analysis

Frozen cultures were thawed on ice and 50 μl of each sample were taken to 800 μl PBS and centrifuged at 4500 rpm for 10 min at 4°C. The supernatants were discarded and the pellets resuspended in 100 μl PBS containing 10 μg ml^-1^ THE^TM^ His Tag [FITC] antibody (Genscript) and 10 μg ml^-1^ Anti-c-Myc [SureLight® Allophycocyanin] antibody (Abcam). The samples were subsequently covered in aluminum foil and incubated at room temperature for 60 min on a tube rotator (Stuart). The samples were then centrifuged (4500 rpm 10 min, 4°C), the supernatants discarded, and the pellets washed in 100 μl PBS. An additional centrifugation followed (4500 rpm 10 min, 4°C) after which the pellets were resuspended in 500 μl ice-cold PBS and transferred to flow cytometer tubes which were kept on ice until analysis on the flow cytometer (Gallios^TM^, Beckman Coulter). 10 000 events were collected for each sample.

## Competing interests

The authors declare that they have no competing interests.

## Authors’ contributions

JJ and MG did the majority of the experiments and analyzed the experimental data. JJ and MG contributed equally to the experimental work. JJ wrote the manuscript. MG contributed to the manuscript. DTH contributed to the experimental work. PS supervised the cloning and contributed to the manuscript. GL was responsible for the original concept, supervised the work and contributed to the manuscript. All authors have read and approved the manuscript.

## Supplementary Material

Additional file 1 DNA sequence for pAIDA1. Click here for file
