# Supplementary material for: A dual tag system for facilitated detection of surface expressed proteins in Escherichia coli
Source: Microb Cell Fact. 2012 Sep 3;11:118. doi: 10.1186/1475-2859-11-118 (PMC3511212; doi:10.1186/1475-2859-11-118)
Supplement: Additional file 1 — DNA sequence for pAIDA1. [file 1475-2859-11-118-S1.pdf]

Supplementary materials 1

DNA sequence for pAIDA1

TGGCACGCGGCGCGCCCCTTGGTGCGCAAACTATTAACTGGCGAACTACTTACTCTAGCTTCCCGG

CAACAATTAATAGACTGGATGGAGGCGGATAAAGTTGCAGGACCACTTCTGCGCTCGGCCCTTCCG

GCTGGCTGGTTTATTGCTGATAAATCTGGAGCCGGTGAGCGTGGGTCTCGCGGTATCATTGCAGCA

CTGGGGCCAGATGGTAAGCCCTCCCGTATCGTAGTTATCTACACGACGGGGAGTCAGGCAACTATG

GATGAACGAAATAGACAGATCGCTGAGATAGGTGCCTCACTGATTAAGCATTGGTAACTGTCAGA

CCAAGTTTACTCATATATACTTTAGATTGATTTAAAACTTCATTTTTAATTTAAAAGGATCTAGGTG

AAGATCCTTTTTGATAATCTCATGACCAAAATCCCTTAACGTGAGTTTTCGTTCCACTGAGCGTCAG

ACCCCTTAATAAGATGATCTTCTTGAGATCGTTTTGGTCTGCGCGTAATCTCTTGCTCTGAAAACGA

AAAAACCGCCTTGCAGGGCGGTTTTTCGAAGGTTCTCTGAGCTACCAACTCTTTGAACCGAGGTAA

CTGGCTTGGAGGAGCGCAGTCACCAAAACTTGTCCTTTCAGTTTAGCCTTAACCGGCGCATGACTT

CAAGACTAACTCCTCTAAATCAATTACCAGTGGCTGCTGCCAGTGGTGCTTTTGCATGTCTTTCCGG

GTTGGACTCAAGACGATAGTTACCGGATAAGGCGCAGCGGTCGGACTGAACGGGGGGTTCGTGCA

TACAGTCCAGCTTGGAGCGAACTGCCTACCCGGAACTGAGTGTCAGGCGTGGAATGAGACAAACG

CGGCCATAACAGCGGAATGACACCGGTAAACCGAAAGGCAGGAACAGGAGAGCGCACGAGGGAG

CCGCCAGGGGGAAACGCCTGGTATCTTTATAGTCCTGTCGGGTTTCGCCACCACTGATTTGAGCGT

CAGATTTCGTGATGCTTGTCAGGGGGGCGGAGCCTATGGAAAAACGGCTTTGCCGCGGCCCTCTCA

CTTCCCTGTTAAGTATCTTCCTGGCATCTTCCAGGAAATCTCCGCCCCGTTCGTAAGCCATTTCCGC

TCGCCGCAGTCGAACGACCGAGCGTAGCGAGTCAGTGAGCGAGGAAGCGGAATATATCCTGTATC

ACATATTCTGCTGACGCACCGGTGCAGCCTTTTTTCTCCTGCCACATGAAGCACTTCACTGACACCC

TCATCAGTGCCAACATAGTAAGCCAGTATACACTCCGCTAGCGCTGAGGTCTGCCTCGTGAAGAAG

GTGTTGCTGACTCATACCAGGCCTGAATCGCCCCATCATCCAGCCAGAAAGTGAGGGAGCCACGGT

TGATGAGAGCTTTGTTGTAGGTGGACCAGTTGGTGATTTTGAACTTTTGCTTTGCCACGGAACGGTC

TGCGTTGTCGGGAAGATGCGTGATCTGATCCTTCAACTCAGCAAAAGTTCGATTTATTCAACAAAG

CCACGTTGTGTCTCAAAATCTCTGATGTTACATTGCACAAGATAAAAATATATCATCATGAACAAT

AAAACTGTCTGCTTACATAAACAGTAATACAAGGGGTGTTATGAGCCATATTCAACGGGAAACGTC

TTGCTCGAGTATCCGCTCATGAGATTATCAAAAAGGATCTTCACCTAGATCCTTTTGTAAGAGGTTC

CAACTTTCACCATAATGAAATAAGATCACTACCGGGCGTATTTTTTGAGTTATCGAGATTTTCAGGA

GCTAAGGAAGCTAAAATGGAGAAAAAAATCACTGGATATACCACCGTTGATATATCCCAATGGCA

TCGTAAAGAACATTTTGAGGCATTTCAGTCAGTTGCTCAATGTACCTATAACCAGACCGTTCAGCT
GGATATTACGGCCTTTTTAAAGACCGTAAAGAAAAATAAGCACAAGTTTTATCCGGCCTTTATTCA
CATTCTTGCCCGCCTGATGAATGCTCATCCGGAGTTTCGTATGGCAATGAAAGACGGTGAGCTGGT
GATATGGGATAGTGTTCACCCTTGTTACACCGTTTTCCATGAGCAAACTGAAACGTTTTCATCGCTC
TGGAGTGAATACCACGACGATTTCCGGCAGTTTCTACACATATATTCGCAAGATGTGGCGTGTTAC
GGTGAAAACCTGGCCTATTTCCCTAAAGGGTTTATTGAGAATATGTTTTTCGTCTCAGCCAATCCCT
GGGTGAGTTTCACCAGTTTTGATTTAAACGTGGCCAATATGGACAACTTCTTCGCCCCCGTTTTCAC
CATGGGCAAATATTATACGCAAGGCGACAAGGTGCTGATGCCGCTGGCGATTCAGGTTCATCATGC
CGTTTGTGATGGCTTCCATGTCGGCAGAATGCTTAATGAATTACAACAGTACTGCGATGAGTGGCA
GGGCGGGGCGTAATTTTTTTAAGGCGACACCATCGAATGGCGCAAAACCTTTCGCGGTATGGCATG
ATAGCGCCCGGAAGAGAGTCAATTCAGGGTGGTGAATGTGAAACCAGTAACGTTATACGATGTCG
CAGAGTATGCCGGTGTCTCTTATCAGACCGTTTCCCGCGTGGTGAACCAGGCCAGCCACGTTTCTG
CGAAAACGCGGGAAAAAGTGGAAGCGGCGATGGCGGAGCTGAATTACATTCCCAACCGCGTGGCA
CAACAACTGGCGGGCAAACAGTCGTTGCTGATTGGCGTTGCCACCTCCAGTCTGGCCCTGCACGCG
CCGTCGCAAATTGTCGCGGCGATTAAATCTCGCGCCGATCAACTGGGTGCCAGCGTGGTGGTGTCG
ATGGTAGAACGAAGCGGCGTCGAAGCCTGTAAAGCGGCGGTGCACAATCTTCTCGCGCAACGCGT
CAGTGGGCTGATCATTAACTATCCGCTGGATGACCAGGATGCCATTGCTGTGGAAGCTGCCTGCAC
TAATGTTCCGGCGTTATTTCTTGATGTCTCTGACCAGACACCCATCAACAGTATTATTTTCTCCCAT
GAAGACGGTACGCGACTGGGCGTGGAGCATCTGGTCGCATTGGGTCACCAGCAAATCGCGCTGTT
AGCGGGCCCATTAAGTTCTGTCTCGGCGCGTCTGCGTCTGGCTGGCTGGCATAAATATCTCACTCGC
AATCAAATTCAGCCGATAGCGGAACGGGAAGGCGACTGGAGTGCCATGTCCGGTTTTCAACAAAC
CATGCAAATGCTGAATGAGGGCATCGTTCCCACTGCGATGCTGGTTGCCAACGATCAGATGGCGCT
GGGCGCAATGCGCGCCATTACCGAGTCCGGGCTGCGCGTTGGTGCGGATATCTCGGTAGTGGGATA
CGACGATACCGAAGACAGCTCATGTTATATCCCGCCGTTAACCACCATCAAACAGGATTTTCGCCT
GCTGGGGCAAACCAGCGTGGACCGCTTGCTGCAACTCTCTCAGGGCCAGGCGGTGAAGGGCAATC
AGCTGTTGCCCGTCTCACTGGTGAAAAGAAAAACCACCCTGGCGCCCAATACGCAAACCGCCTCTC
CCCGCGCGTTGGCCGATTCATTAATGCAGCTGGCACGACAGGTTTCCCGACTGGAAAGCGGGCAGT
GAGTGGATAACCGTATTACCGCCTTTGAGTGAGCTGATACCGGGAATTCTCACTCATTAGGCACCC
CAGGCTTTACACTTTATGCTTCCGGCTCGTATAATGTGTGGAATTGTGAGCGGATAACAATTTCACA
CAGGAAAGCTTCATATGAATAAGGCCTACAGTATCATTTGGAGCCACTCCAGACAGGCCTGGATTG

TGGCCTCAGAGTTAGCCAGAGGACATGGTTTTGTCCTTGCAAAAAATACACTGCTGGTATTGGCGG

TTGTTTCCACAATCGGAAATGCATTTGCAGTCGACCACCATCACCATCACCATCTGGAAGCGCTGTT

CCAGGGTCCGGGTACCCAGAAACAGCGTACCGAGCTCGAAAACCTGTACTTCCAGGGTGAACAGA

AACTGATTAGCGAAGAAGATCTGTCTAGAGTGAATAACAATGGAAGCATTGTCATTAATAACAGC

ATTATAAACGGGAATATTACGAATGATGCTGACTTAAGTTTTGGTACAGCAAAGCTGCTCTCTGCT

ACAGTGAATGGTAGTCTTGTTAATAACAAAAATATCATTCTTAATCCTACAAAAGAAAGTGCGGCC

GCTATAGGTAATACTCTTACCGTGTCAAATTATACTGGGACACCGGGAAGTGTTATTTCTCTTGGTG

GTGTGCTTGAAGGAGATAATTCACTTACGGACCGTCTGGTGGTGAAAGGTAATACCTCTGGTCAAA

GTGACATCGTTTATGTCAATGAAGATGGCAGTGGTGGTCAGACGAGAGATGGTATTAATATTATTT

CTGTAGAGGGAAATTCTGATGCAGAATTCTCTCTGAAGAACCGCGTAGTTGCCGGAGCTTATGATT

ACACACTGCAGAAAGGAAACGAGAGTGGGACAGATAATAAGGGATGGTATTTAACCAGTCATCTT

CCCACATCTGATACCCGGCAATACAGACCGGAGAACGGAAGTTATGCTACCAATATGGCACTGGCT

AACTCACTGTTCCTCATGGATTTGAATGAGCGTAAGCAATTCAGGGCCATGAGTGATAATACACAG

CCTGAGTCTGCATCCGTGTGGATGAAGATCACTGGAGGAATAAGCTCTGGTAAGCTGAATGACGG

GCAAAATAAAACAACAACCAATCAGTTTATCAATCAGCTCGGGGGGGATATTTATAAATTCCATGC

TGAACAACTGGGTGATTTTACCTTAGGGATTATGGGAGGATACGCGAATGCAAAAGGTAAAACGA

TAAATTACACGAGCAACAAAGCTGCCAGAAACACACTGGATGGTTATTCTGTCGGGGTATACGGTA

CGTGGTATCAGAATGGGGAAAATGCAACAGGGCTCTTTGCTGAAACTTGGATGCAATATAACTGGT

TTAATGCATCAGTGAAAGGTGACGGACTGGAAGAAGAAAAATATAATCTGAATGGTTTAACCGCTT

CTGCAGGTGGGGGATATAACCTGAATGTGCACACATGGACATCACCTGAAGGAATAACAGGTGAA

TTCTGGTTACAGCCTCATTTGCAGGCTGTCTGGATGGGGGTTACACCGGATACACATCAGGAGGAT

AACGGAACGGTGGTGCAGGGAGCAGGGAAAAATAATATTCAGACAAAAGCAGGTATTCGTGCATC

CTGGAAGGTGAAAAGCACCCTGGATAAGGATACCGGGCGGAGGTTCCGTCCGTATATAGAGGCAA

ACTGGATCCATAACACTCATGAATTTGGTGTTAAAATGAGTGATGACAGCCAGTTGTTGTCAGGTA

GCCGAAATCAGGGAGAGATAAAGACAGGTATTGAAGGGGTGATTACTCAAAACTTGTCAGTGAAT

GGCGGAGTCGCATATCAGGCAGGAGGTCACGGGAGCAATGCCATCTCCGGAGCACTGGGGATAAA

ATACAGCTTCTGATAATGATCC
